# Complex strategies: an integrative analysis of contests in Siamese fighting fish

**DOI:** 10.1186/s40850-022-00156-3

**Published:** 2022-12-09

**Authors:** Kyriacos Kareklas, Hansjoerg P. Kunc, Gareth Arnott

**Affiliations:** 1 Integrative Behavioural Biology Group, Instituto Gulbenkian de Ciência, Rua da Quinta Grande 6, 2780-156 Oeiras, Portugal; 2 School of Biological Sciences, Queen’s University Belfast, 19 Chlorine Gardens, Belfast, Northern Ireland, BT9 5DL UK

**Keywords:** Contest, Strategies, Decisions, Motivation, RHP, Resource value, Assessment

## Abstract

**Background:**

Animals use contests to attain resources and employ strategic decisions to minimise contest costs. These decisions are defined by behavioural response to resource value and competitive ability, but remain poorly understood. This is because the two factors are typically studied separately. Also, their study relies on overgeneralised assumptions that (i) strategies are fixed, (ii) modulated by the motivation or drive to fight and (iii) used to manage costs proportional to the timing of the loser’s retreat. To address these problems, we adopt an integrative sequential analysis that incorporates competitive ability and resource value factors, to characterise territorial contest decisions in male Siamese fighting fish (*Betta splendens*).

**Results:**

Individuals exhibited a chronological organisation of behaviour, engaging opponents first with frontal display, then switching to lateral display before deciding to attack, and reserved retreats for later stages. Using asymmetries in retreats as a proxy for outcome, the likelihood of winning was found to be mostly dependent on display. However, resource and contest conditions affected initiation latency, display, attack and retreat, suggesting that strategic decisions influence all behaviour. Overall, sequential behaviour varied consistently with individual aggressiveness and resource-value factors, and increasingly with information on competitive ability collected during the contest. This enabled shifts in tactics, such as disadvantaged individuals responding first with aggression and later with submission. Motivation to continue fighting, after interruption by startle, was also adjusted to information gathered during the contest and progressively with energetic state. Two clusters of correlated behaviours were identified, cost-mitigation (display and retreat) and escalation (initiation and attack), but changes in motivation were associated only with cost mitigation.

**Conclusions:**

Our findings contrast dominant assumptions that strategic decisions are fixed, controlled by motivational state and sufficiently described by outcome-dependent measures. We instead demonstrate that strategic decisions are complex, comprising functional changes in assessment, information use and motivational effects, which are not always inter-dependent.

**Supplementary Information:**

The online version contains supplementary material available at 10.1186/s40850-022-00156-3.

## Background

Contests are used to attain resources, but can impose high energetic and injury costs [1]. According to theory, these costs can be managed by strategic decisions regarding the resource’s value and the contestants’ fighting ability, termed resource holding potential (RHP) [2, 3]. Thus, while winning contests enables access to resources, contestants may adopt strategic decisions to modulate behaviour during contests to minimize the costs [1–5]. Most studies of strategic decisions tend to focus on either resource value or RHP assessment rules, determining strategies in terms of whether contest duration or outcome relies on one’s own capacities or also on the opponent’s, be it morphology or behaviour [2–7]. Furthermore, with some rare exceptions [8–10], studies on agonistic interactions consider that the decisions made by individuals do not change throughout the contest. Here, we adopt a more comprehensive or generalised definition of strategic decisions, involving decisions made by rivals at different moments of the contest that may reduce the contest costs based on updated information on both resource value and RHP, while also accounting for effects from individual aggressiveness.

The characterisation of contest strategies has been limited by the lack of integration of resource value and RHP assessment, and accounting for behavioural variation between individuals. However, suggestions for new generalised approaches to address this (e.g. repeated-trial and ternary-plot frameworks), continue to rely on a set of assumptions that have dominated the field regarding the assessment of information used in strategic decisions and the behavioural outputs of these decisions [4, 5]. Specifically, expectations that only a fixed set of information is collected, that a particular assessment type is involved and that strategic decisions can be ascertained by changes in contest duration and outcome, are increasingly being disputed [6–11].

The first set of assumptions regards the assessment of resource value, as first posited by Parker and Stuart in their 1976 model [12]. In particular, effects on strategic decisions are often expected to rely on information solely on the objective quality of the resource in terms of intrinsic characteristics, such as the size of contested shells in hermit crabs [11], and extrinsic environmental conditions, such as water flow in anemone territorial fights [6]. However, resource value can vary ‘subjectively’ based on life-history, such as territorial ownership or prior nesting [2]. Thus, decisions regarding resource value may depend not only on perceived quality or subjective value, but an interaction between both, as demonstrated in parasitoid wasps by Storckermans and Hardy [13].

The second set of assumptions regards RHP assessment, where theory predicts that this involves fixed strategies of either self, opponent or mutual assessments of morphology and behaviour, or cumulative assessments of energetic or injury thresholds [3, 7]. Either of these strategies could be employed sequentially during contests, but there is also rare evidence of shifts in the form of assessment across sequential phases, such as the shift from mutual size assessment during display to that of self-assessment during escalated attacks in killifish [8]. This suggests that decisions may vary by incorporating new information as the contest progresses, and this may implicate various forms of assessment [9, 10]. Therefore, the assumption that decisions rely on one particular form of assessment, e.g. only self or mutual assessment, or that strategies are fixed, i.e. repeatedly involve a specific set of information that limits the improvement of estimates in contest costs, may not always hold.

Another set of assumptions regards the way by which strategies have historically been determined by variations in contest duration. A key prediction about strategic decisions is that weaker individuals lose and that their decision to quit is faster when resource value is low, when they have low contest abilities, or when their opponent is stronger, to mitigate energetic or injury costs [2, 3]. As such, strategic decisions have been tested by examining the relationship between contest duration, as determined by the loser’s retreat, and the winner’s and loser’s RHP, or RHP disparity, such as in body and weaponry size [3, 7]. This has overwhelmingly framed research in terms of outcome, while the likelihood for alternative strategies is often overlooked. For instance, weaker individuals can manage costs or win via dishonest display signals of fighting abilities (e.g. cheliped size in hermit-crabs [11]) or via indirect damage to the opponent’s RHP (e.g. sabotage of ornaments in bowerbird mate contests [14]), or contestants may decide to attack even if this is more injurious to themselves than their opponents [15]. Thus, the focus on outcome relies on overgeneralised assumptions because strategic decisions primarily affect behaviour, such as display, attack and retreat tendencies, which comprises much greater variation than that described by contest duration or by the binary attribute of winning or losing [16–18].

Moreover, because a contest’s duration is determined by the timing of the loser’s decision to quit, it is often assumed to reflect their fight motivation, i.e. the drive to continue fighting. This assumption has been criticised because contest duration also relies on ongoing behaviour, and is thus not an independent measure of motivation [19, 20]. Nevertheless, motivation can be susceptible to strategic decisions during contests but also involved in the modulation of behaviour, such as escalation or retreat [2, 19]. Because of this, behaviour is often assumed to directly reflect motivational changes determined by resource benefits and fight costs [21]. However, motivational effects are often difficult to disentangle from neurocognitive components and motivation does not always rely on cost-benefit assessments nor does it affect all behaviour [22]. For example, contestants can be highly motivated to fight for a key resource irrespective of their opponent’s size and their motivation may relate variably to different display and attack behaviours [11, 20]. Thus, the involvement of motivation in strategic decisions cannot be assumed from the relationship between behaviour and RHP or resource factors. Instead, it is a likely contributor of behavioural variation whose susceptibility to factors and its degree of contribution require quantification in any given situation.

Another contributor to behavioural variation is baseline individual differences in aggressiveness, whose effects on strategic decisions are underexplored [16–18]. Aggressiveness contributes individual phenotypic variation in different behavioural tendencies, such levels of display and attack. These tendencies can exhibit relationships between them and some degree of stability, i.e. consistency in inter-individual variation across time and conditions. For example, in green swordtails, more aggressive individuals consistently exhibit more rigorous display or attack across contests with differently sized rivals [16]. These consistent individual differences are expected to have an underlying impact on behavioural variation on top of which added variation due to decisions occurs [17, 18]. However, these consistent effects may also potentially limit the extent to which behaviour can be modulated by strategic decisions. In particular, if aggressiveness predicts most behavioural variation consistently during contests, then the ability of animals to exhibit added behavioural adjustments to new information is restricted. Thus, an important knowledge-gap is whether the contribution of individual aggressiveness to behavioural expressions is as stable as assumed or if it is traded off during contests for modulation by progressively collected information.

**Fig. 1:**
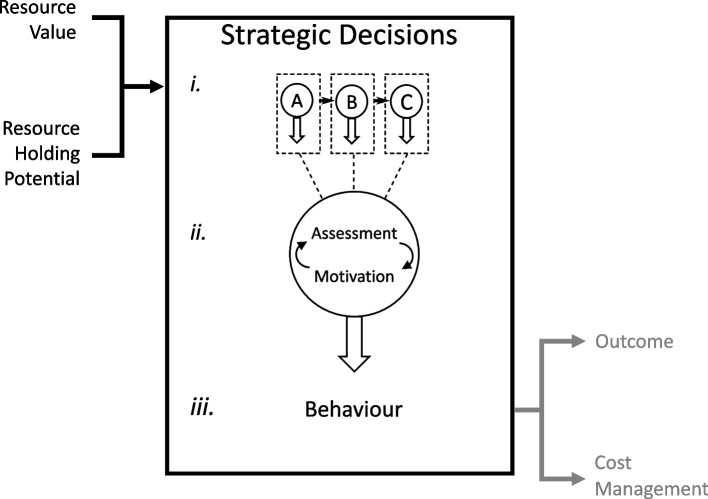
Characterising strategic decisions. Research usually focuses on either resource value or RHP and their influence on outcome and cost management, e.g. faster retreat by weaker animals. These effects are assumed to rely on the fixed use of assessment strategies, and their effect on behaviour is considered an expression of the contestants’ drive to fight, i.e. their motivation. To characterise the complexity of strategic decisions we argue that integrative studies of the combined effects from RHP and resource value should explicitly test these assumptions by examining (i) progressive changes in decisions and (ii) the inter-play between motivation and assessment, and by (iii) characterising all behavioural output instead of only contest duration and outcome

The combined evidence supports the need for an integrative approach that examines the influence of both resource value and RHP assessment together, as well as trade-offs with individual aggressiveness. Yet, we further stress that the full complexity of strategic decisions requires testing assumptions about their (i) stability, (ii) the implication of motivation and (iii) the resultant variation of behaviour (Fig. 1). Here we address this by using an integrative sequential analysis of behaviour during territorial contests in male Siamese fighting fish, *Betta splendens*. These contests play a significant role in reproductive success due to the use of territories to build bubble-nests for their offspring, which contributes fundamentally to the survival and development of eggs and prepares males for mating [23, 24]. Thus, a territory has great fitness value that can drive highly aggressive behaviour both by males looking to build nests and those protecting existing ones [23–27]. This aggressiveness underlies the lengthy, physically taxing and often deadly interactions necessary to resolve contests. Given the severity of these contests, when conducting research, interactions are typically staged in a way that prevents direct physical contact, and of limited duration, with winners determined by proxy-behaviours or behavioural criteria [28]. This predetermines that contest outcome is dependent on behaviour, but with little consideration of the likely added role of behaviour to manage contest costs that exceed the loss of a fight, such as injury and mortality risks. Such strategic decisions by *B. splendens*, whether for wining or minimizing costs, involve a well-documented, stereotyped and easily identifiable behavioural repertoire [29, 30]. This includes frontal displays with extended gill-covers and lateral displays with spread fins for signalling size, biting and tail-beating attacks for inflicting physical injury, and retreats for avoiding attack or signalling submission [23–31]. Even though evidence is rare, these behaviours have been noted to exhibit chronological organisation during contests and individual variation due to underlying personality-trait aggressiveness [31, 32]. In addition, the behaviours can be adjusted to assessments of RHP and the perceived value of defended territory, which can vary in quality with noise disturbances and ‘subjectively’ with the construction of bubble-nests [23–25]. Finally, recovery from mid-contest startles has been repeatedly validated as an independent measure of fight motivation across species, including in *B. splendens* where it exhibits similar but different strategic modulation to behaviour [20, 24, 25, 33]. Hence, using this system we quantify strategic decisions in terms of integrated effects of resource value, RHP and individual aggressiveness on progressive contest behaviour, as well its interplay with motivation.

## Results

**Fig. 2:**
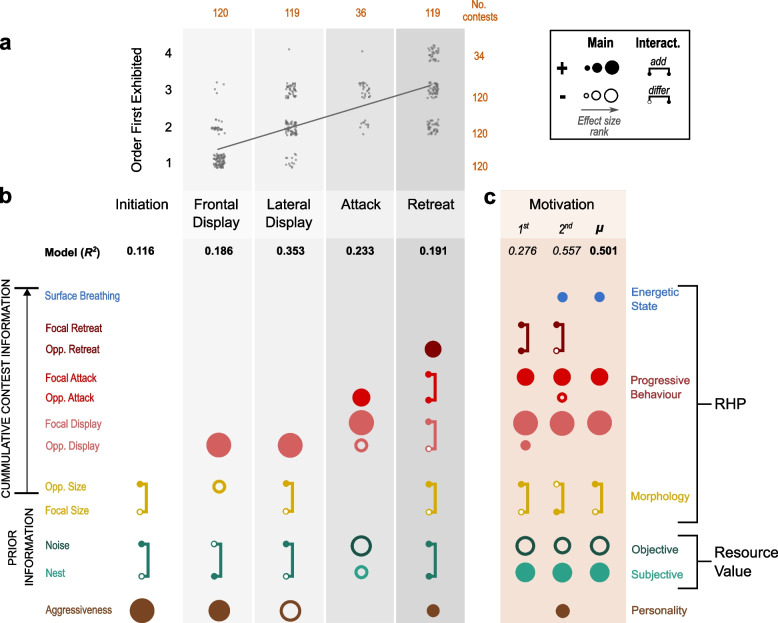
Progressive changes in contest phenotype and its adjustment to assessment-based decisions. (**a**) Based on the chronological order in which animals first exhibited each behaviour, across contests there was a strong preference for a particular sequence of behaviours. (**b**) The contribution of consistent predictors, such as trait aggressiveness, resource value factors and morphological measures, varied across sequential outputs, and progressive effects by preceding behaviour were identified. (**c**) Motivation was also adjusted by resource value, morphology and behavioural information, but was also progressively affected by own energetic state [all models were statistically significant at *P* < 0.001]

Contest behaviours were repeatedly adopted across the staged 120 contests, except attack which was expressed by focal fish in only 36 contests (Fig. 2a). The chronological order in which separate behaviours tended to be first exhibited was significantly different (*R*^*2*^ = 0.601, χ^2^_3, 393_ = 80.87, *P* < 0.001), with a prevalence for frontal display preceding lateral display, followed by any attack, and retreat reserved typically at later stages but also as early as second (Fig. 2a). This is consistent with the predicted function of different behaviours, such as the use of displays to dissuade opponents before attack and later use of retreats to escape attacks or signal submission.

Based on whether opponents retreated more than focal fish, the likelihood of winning was only weakly predicted by behaviour (model: *R*^*2*^ = 0.066, *χ*^*2*^_*2, 119*_ = 10.72, *P* = 0.005), having positive effects from total display duration (*β* = 0.002, *χ*^*2*^_*1,119*_ = 5.70, *P* = 0.017) but negative effects from attack (*β* = − 0.986, *χ*^*2*^_*1,119*_ = 5.12, *P* = 0.024). However, when quantifying the modulation of all behavior, after controlling for random individual effects (*R*^*2*^_*C*_ = 0.409), models of resource value, RHP, and individual aggressiveness predicted much of the combined variation in behaviour (*P* < 0.001; *R*^*2*^_*C*_ = 0.239). Resource value factors (22.37% FEV), trait aggressiveness (16.30% FEV) and morphological factors (9.65% FEV) had repeated effects, but the greatest contributor was progressively available information from ongoing behaviour during contests (43.22% FEV).

Contributions by each predictor across phenotypic measures are summarised in Fig. 2 (b and c) and detailed in Table S1 (Additional file 1). In general, more aggressive animals started contests faster (*β* = 0.4) and retreated slightly less (*β* = 0.1), preferring frontal (*β* = 0.2) to lateral display (*β* = − 0.2). High resource value from nested territory without noise (interaction) drove faster initiation (*β* = 0.6), preference for frontal display (*β* = 0.1) and reduced retreat (*β* = − 0.3), while both nests and noise suppressed attacks (*β* ≤ − 1.2). Against relatively bigger opponents (interaction), fish started contests faster (*β* = 0.3), but also retreated more (*β* = 0.3), preferring lateral display during contests (*β* = 0.3). In terms of progressive behaviour, individuals matched their opponent’s response in display, attack and retreat (*β* ≥ 0.2), but attacks were also exhibited by fish with more rigorous display (*β* = 0.7) and retreat was more likely when opponents attacked more (*β* = 0.4) and displayed less (*β* = − 0.1) than focals. Motivation was consistently higher when defending nested territory (*β* ≥ 0.5) and lower when there was added noise (*β* < − 0.3). It was also greater for animals with a size advantage (*β* ≥ 1.3), and who displayed (*β* ≥ 0.2) and attacked (*β* ≥ 0.6) more, and retreated less than their opponents (interaction; *β* ≥ 0.1). Earlier in the fight, motivation was elevated against opponents that displayed more (probe 1, *β* = 0.1). However, later in the fight (probe 2), motivation was instead reduced if opponents attacked (*β* = − 0.5) and higher for fish with greater rates of surface breathing (*β* = 0.2).

**Fig. 3:**
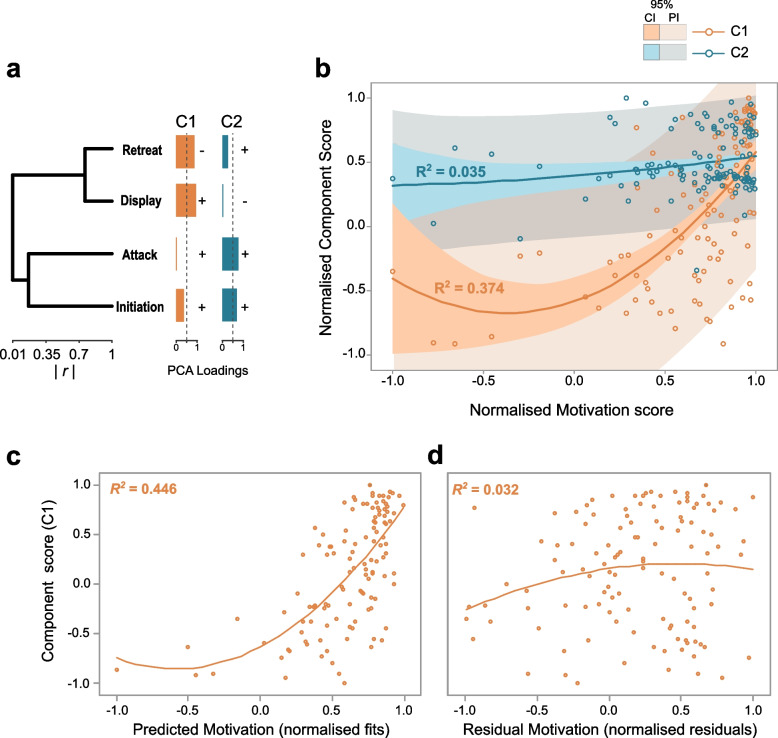
Phenotypic architecture of contest strategy. (**a**) Based on inter-behaviour correlations, two separate modules were revealed, represented first by absolute correlation clusters (*| r* |), and quantified via PCA in two discrete components, one comprising mainly variation in behaviours of *cost mitigation* (C1, orange bars) – display and retreat – and another comprising mainly behaviours of contest *escalation* (C2, green bars) – initiation and attack tendency. (**b**) Only the first component related significantly to motivational state (average recovery from startle probes) and (**c**) the effect was reserved mostly for the variation in motivation predicted by the RHP, resource value and personality factors in the model (fits), compared to (**d**) the residual variation. Units are of normalised scores

Based on correlations, behaviour during contests was categorised in two distinct modules and reduced by PCA (eigenvalue > 1) to two corresponding discrete components (Fig. 3a). By comparing loadings against the recommended criterion of > 0.5 [34], the first component (C1: *% Var* = 44.7) accounted chiefly for display (0.941) and retreat (− 0.876), whereas the second component (C2: *% Var* = 27.4) accounted for initiation (0.668) and attack (0.760). Average motivation predicted the first (*F*_*1119*_ = 65.17, *P* < 0.001), but not the second component (*F*_*1119*_ = 2.10, *P* = 0.150) (Fig. 3b). The variation of average motivation predicted by the model (fits: *F*_*1119*_ = 79.87, *P* < 0.001) had markedly greater effect than residual variation (*F*_*3119*_ = 4.65, *P* = 0.033) (Fig. 3c).

## Discussion

Contest strategies are typically defined as behavioural changes tied to outcome, i.e. winning or losing, and are considered to rely on fixed sets of information, on particular forms of assessment and mediated by motivational state [2, 3, 21]. However, our integrative approach reveals that, while outcome was weakly predicted by behaviour, behaviour itself was largely affected by baseline individual variation and strategic flexibility from the use of progressively available information and mixed assessment tactics. This included resource and mutual-size assessments for the decision to initiate fights, which were progressively complemented by opponent-only assessments for matching behaviour, and ultimately included mutual assessments of behaviour affecting attack and retreat, while motivation in later stages relied on self-assessment of energetic thresholds. This is facilitated by the chronological organisation of contest behaviour and the sequential adjustment of both behaviour and motivation (Fig. 2). We also show that contest behaviour is structured in functionally discrete components and which rely differentially on motivational state and its modulation by strategic decisions (Fig. 3).

### Sequential organization of behaviour

The chronological organisation of contest behaviour by male *B. splendens* (Fig. 2a) includes the early use of displays, which is consistent with their use to signal size and aggressive intent for resolving contests without escalation [15, 31, 35]. This has been previously observed in *B. splendens*, but is also common in other vertebrates and in invertebrates, such as horses and fiddler crabs [27, 36, 37]. The preference for frontal display to initiate contests is regarded as an acute immediate response followed by the less energetically costly lateral display [31]. Attack tended to follow display, but the majority of contests relied on display without attack, which demonstrates directly that display mitigates escalation [15, 35]. Retreats tended to be reserved for later stages of the fight, which suggests that it is used to briefly escape an opponent attack or signal submission. Yet, animals also exhibited retreat as early as second in order and followed by either display or attack. Together with their short duration, repeated use and never being exhibited as full withdrawal at the end of the fight, these retreats were not indicative of quitting or losing [38, 39]. Instead, they might be employed as a temporary withdrawal tactic in response to assessed advantages in opponent size or behaviour (Fig. 2c), which enables the mitigation of potential or imminent costs. In the wild, males may use their early and intense expression of display signals for claiming territory and defending nests from multiple potential intruders at the same time, while the sequential organisation enables them to progressively incorporate information from preceding behaviour, both theirs and their opponent’s, when deciding to abandon these resources (Fig. 2c).

### Cumulative information and the progressive adjustment of motivation

The consistent effects of nest presence and noise on agonistic behaviour and motivation indicate the overarching significance of resource value. Nests contribute to mating readiness and offspring survival, but noise can reduce perceived territorial value [23, 24]. Thus, the negative interaction between noise and nest on initiation and display signifies that decisions to fight rely on comparative differences designating a balanced value from costs against benefits. This mechanism ensures that costly fights in the wild are carefully employed only when a resource’s value is exceedingly high, which poses questions about competition in *B. splendens* natural environments that are subject to extensive human disturbance, such as rice paddies [23]. Paradoxically, fish exhibited more lateral display under noise and less in the presence of nests, which suggests that it is preferred during fights over low value resources. This is possibly due to lateral display being less energetically costly than frontal display and, thus, preferable for low-gain fights [31]. Attack was not affected by noise-nest interactions and was instead inhibited by both factors. In the case of noise this may be due to low territorial value, but in the case of bubble nests it may relate either to low energetic reserves from prior nest-building or to the demanding prospects of future reproductive investment [23]. However, positive interaction effects on retreat show that under noise fish with nests retreated more, possibly due to uncertainty, but further study of this is warranted [24, 40].

Individual aggressiveness also contributed consistently to behaviour, as expected for baseline personality differences [16–18], but its effect size was progressively traded-off with the increasing size of RHP effects (Fig. 2b). The low lateral display by more aggressive fish and the lack of effects on attack contrast expectations of stability in the behaviour exhibited repeatedly during the pre-fight mirror tests. This could be due to strategic adjustments that are not sufficiently represented by mirror tests compared to real-opponent contests [30]. Nevertheless, the implication of individuality is consistent with previous findings and in the wild may be used by third-party observers to recognize dominant opponents and reduce the need for future fights. This is supported by evidence that *B splendens* can communicate individualities in their behavior [32] and that these individualities can be recognized by others that had previously observed them as audience to third-party aggressive interactions [29].

Morphology had unpredictable effects and its assessment elicited different tactics, including mutual comparisons (Fig. 2b). On the one hand, faster initiation and increased lateral display towards relatively bigger opponents is consistent with previously demonstrated effects in *B. splendens*, a response often attributed to either the resource-dependent “Napoleon strategy” or the nothing-to-lose “desperado effect” [41]. On the other hand, the elevated tendency to retreat when facing bigger opponents reveals submission to the opponent’s superiority as predicted by theorised cost-mitigation strategic decisions [3]. Together, findings suggest that the two tactics were co-opted for a mixed strategy of early aggression and later submission. Frontal display was overall preferred against smaller opponents, likely due the low necessity to use lateral displays of body and fin to ascertain size advantage, while attack was unaffected by morphology assessments. Tail beating is one of the two attacks used *by B. splendens.* The potential effectiveness of this motor-dependent behaviour may be better assessed from movement during displays than extrapolated from the size of the opponents, which was instead mutually assessed (Fig. 2b).

Fish also consistently assessed and matched their opponents display, attack and retreat. Yet there was also a progressive increase in the use of mutual behaviour information, where attack was affected by both focal and opponent behaviour, and retreat by their interaction. However, where the interaction effect between focal and opponent display was negative, revealing the use of comparative differences [3, 7], the interaction effect between focal and opponent attack was positive, revealing that retreats could follow both being attacked and attacking. This suggests that attacks are not necessarily used to impose dominance over opponents and this is consistent with our finding that escalated attacks decrease the likelihood of winning. Although, this could be an artefact of estimating winning likelihood from asymmetries in retreats, which may have been used for injury avoidance and not to submit.

Motivational state was also dependent on resource value and mutual assessments of both morphology and behaviour, but progressively was also positively affected by air-breathing rates. Increases in surface air-breathing are exhibited by *B. splendens* when performing more rigorous display and attack, providing the energetic reserves to sustain such demanding behaviour [30]. This suggests that greater energetic reserves maintain higher levels of motivation. Yet, on the flip side this means that low energetic reserves drive lower motivation levels. As such, the later-stage effect of air-breathing indicates the cumulative assessment of energetic costs theorised by some self-assessment models and demonstrated by decisions to quit in some species [35, 42].

The progressive integration of information enables improvements in decision accuracy, as previously suggested for *B. splendens* [25]. This may rely on the simultaneous adjustments in motivation or simply on functional differences between behaviours, which require particular forms of assessment to manage cognitive demands and relevant trade-offs between speed and accuracy [22, 43]. The most accurate decisions are derived from the comparison of own to opponent ability [2], which was used regularly for the assessment of size and behaviour. Yet, assessments of behaviour also included opponent-only assessment for matching and self-assessments of energetic state, which require less information gathering and enable more timely responses to opponents and energetic thresholds.

### Phenotypic architecture of contest strategy

Behaviour clustered between two main components (Fig. 3a), each characterised by different strategic function. In particular, display is a tactic aimed at signalling RHP advantages and discouraging weaker opponents early in the contest without risking injury or energy costs from longer or escalated interactions [15, 25–28, 35]. Similarly, retreats are adopted to mitigate injury or energy costs by signalling submission and promoting de-escalation [15, 44]. Thus, the clustering of these two behaviours defines the first strategic component as a *cost mitigation* one. In turn, faster initiation of fights and the decision to attack both comprise the shift to more costly activity and their clustering designates the second strategic component as an *escalation* one [15, 45].

According to some theory, shifts between these two components relate to cost and benefit contributions from RHP and resource value [2, 3]. However, instead of complex decision-making processes, this may be conceptualised as a two-dimensional space state, as proposed by Elwood and Arnott [21], where shifts in the expression of the two components is modulated by motivational effects from resource value on one dimension and RHP on a second dimension. In that model, motivation is considered implicit via the effects on behaviour across the two-dimensional space. However, here we measure this directly and reveal that it is partly true and specific to the cost-mitigation component (Fig. 3b), where effects are largely due to RHP and resource value contributions, as described by our model of average motivation (fits; Fig. 3c). In comparison, residual variation in motivation had a considerably smaller effect on this component (Fig. 3d). The lack of motivational effects on the escalation component suggests that it relies on direct assessment-based decisions revealed in our models of initiation and attack. This might benefit individuals when decisions involve the increasing likelihood of injury and energetic cost, and motivational drives can undermine the ability to accurately estimate these costs. In simpler terms, the motivation to defend resources drives responses meant to achieve this in the least costly manner, but responses with costlier consequences rely only on informed decisions. Although this may not be true for other species, such as for cichlids where motivation can drive both display and attack [33], our results show that motivational effects should be quantified and not assumed for all behaviour or generalised across species.

## Conclusions

We present an integrative approach by which contest strategies can be better characterised via the comprehensive analysis of behavioural changes. By doing so we identify baseline personality effects, stable resource value contributions and functionally varied sequential RHP assessments in *B. splendens*. Amongst these we reveal unexpected directional effects, where trait aggressiveness affects fight initiation and display more than it does escalation and submission, and individuals may increase the use of some display behaviours when defending low quality territory or may shift early-contest aggression to later-stage submission when fighting bigger opponents. Moreover, we find that overall sequential assessments impact both behaviour and motivation by integrating new information on progressive behaviour and energetic state. Finally, we address the question of whether assessments influence motivational state and demonstrate that motivation modulates behaviour following the assessment of RHP and resource value, but only partly. This is because costly escalation-type responses, such as contest initiation and physical attack, rely on information-based decisions without significant motivational effects. Our findings demonstrate the complexity of contest strategies and argue for the future use of integrative behavioural studies for quantifying these complexities.

## Methods

### Animals and housing

Commercially acquired adult *B. splendens* males (*N* = 56) were housed individually in 15 L tanks environmentally enriched with plastic plants, shelters and surface platforms (filtered and aerated; 26 ± 1 °C; 7.2 ± 0.4 pH; 750 μS/cm), kept under 12 h photoperiods (0700–1900, 300 lx) and fed a high-protein diet (Hikari© Bio-gold; 8 pellets/day).

### Experimental protocol

We tested 30 focal animals using a previously validated within-individual repeated measures protocol comprising four weekly contests against a bigger and smaller opponent (relative weight) under noise and control conditions (cf Kareklas et al. [24]). Briefly, focal tanks were covered by a soundproofed lid and during treatment conditions played white noise that elicited changes in underwater acoustic profiles (20–40 dB) ranging within the species hearing thresholds (≤ 5 kHz). Territorial intrusions were staged via between-tank interactions with neighbouring stimulus fish (*n* = 26; acclimated overnight). Following the first agonistic behaviour by focals, we allowed 15 min interactions separated in 5 min intervals framed by two motivational probes via the validated startle approach, i.e. a distinct splash from a drop of marble visually hidden from opponents (cf Kareklas et al. [24, 25]). We used video recordings (*Sony HDR CX190E*) to score behaviour via the Observer XT 11.5 software (Noldus Information Technology).

### Aggressiveness trait

We quantified aggressiveness before the staged contests via the repeated measurement of behaviour (initiation latency, total display time and attack frequency) on two separate instances (housing day 4 and 7), using the mirror test. Although the mirror test is not always representative of intraspecific aggression, it adequately quantifies *B. splendens* aggressive behaviour and controls for carry-over learning effects [10, 30]. Composite aggressiveness scores at each test were extracted via Principal Components Analysis (PCA) on the correlation matrix of behavioural measures (Table S2, Additional file 2; KMO > 0.5; Bartlett’s, *P* < 0.05; *ρ* > 0.6 [34]) and were found stable between day 4 and 7 (*r* = 0.575, *F*_*1,29*_ = 2.35, *P* = 0.012), indicating an aggressive tendency which we quantified by the mean score (PCA Aggressiveness Data, Additional file 3). To control potential multicollinearity effects with size measures, which we intended to also use as predictors of staged-contest behaviour, we tested the relationship of mean aggressiveness score with weight and found a negative but non-significant correlation (*r* = − 0.250, *P* = 0.183).

### Resource value measures

To manipulate subjective value, individuals were housed in their enriched tanks for two weeks before experiments, to allow territorial establishment and the opportunity for construction of bubble-nests. Thus, territorial value primarily varied in terms of the presence or absence of nests in established territories. Added variation in resource quality was manipulated by acoustic conditions during contests, varying between ambient controls and a validated noise treatment localised to focal fish, which according to previous evidence negatively affects resource value by reducing territorial defence and nesting [24]. The treatment comprised playbacks of white noise (low-pass to 100 Hz, 6 dB kHz^− 1^ decrease) induced an underwater 20–40 dB change in sound pressure levels, at frequencies of 1–4 kHz, similar to noise imposed by light human traffic. Manipulations of background noise were initiated 10 min before the staged contests to allow time for focal fish to assess the acoustic changes in their territory.

### RHP factors

#### Morphology

For both focal individuals and their opponents, we used wet weight as a composite measure of morphological state that strongly predicts visually available information, including standard length (*r*^*2*^ = 0.549; *F*_*56*_ = 65.76, *P* < 0.001) and fin size (*r*^*2*^ = 0.445; *F*_*56*_ = 43.31, *P* < 0.001).

#### Behaviour

We recorded ongoing behaviour that can be observed in opponents and/or compared to own behaviour in terms of display intensity, escalation and submission, i.e. rates of frontal gill-flaring and lateral fin-flaring display, occurrence of escalated attacks (biting or tail beating attempts) and rate of retreats respectively (Model Data, Additional file 3). Also, to test the use of cumulative-type self-assessments of energetic state we measured rates of focal air breathing, which is used by *B. splendens* to compensate for higher energetic expenditure during contests [3, 30].

### Behavioural characterisation

For characterising contest strategy, we quantified six phenotypic outputs (Model Data, Additional file 3). Contest *initiation tendency*, an indicator of preparedness to engage opponents and a signal of aggression, was measured by the additive inverse of the time taken to exhibit their first aggressive act against opponents (i.e. their negative latency). *Motivational state* was measured at the two separate probes during the contest and quantified by the average recovery rate from startles (negative duration of motionless response [20, 24, 25, 33]). Finally, we measured four behaviours: *frontal display* of extended gill covers, an energetically costly display of aggression and size [31], *lateral display* of honest and dishonest signals of body size, including length and spread fins [25], and *retreat*, or withdrawal from opponents, all scored by total duration (in seconds), and any biting or tail beating attempts [26] scored as escalated *attack* occurrence (Y/N).

### Analysis

For our analyses we used mixed regression models with a log-link function for non-parametric continuous measures, a binary logistic function for binary data and an ordinal regression with a logit function for ordered data. We included individual identity as a random factor and removed non-significant interaction terms. We quantified predictor reliability by coefficients of determination (*R*^*2*^) derived from the proportion of explained over total variance following the scaled deviance method, where deviance is considered identical to the residual sum of squares [46]. Effect size was measured by standardised *β* coefficients. All analyses were performed using the statistical software SPSS (version 22; IBM Corp., Armonk, NY. USA) and Minitab® (version 17; Minitab Inc., State College, PA, USA).

In order to quantify the contribution of behaviour during contests to outcome, we characterised winning in focals with lower retreat rates than opponents (Y/N), and tested if the likelihood related to initiation, display or attack (binary). In order to provide an integrative sequential analysis (i.e. composite resource-value, RHP and aggressiveness effects on progressive behaviour), we followed three steps. First, sequential organisation of behaviour was ascertained by testing for differences in the chronological order in which separate contest behaviours were first exhibited, i.e. rank in the latency to first exhibit each behaviour (ordinal with Log-Likelihood Ratio Test). Second, for each behavioural measure we tested for effects by models (log-link) that integrated our three *predictor categories*: individual aggressiveness trait (score), resource value (noise and nest) and RHP factors (weight and progressive behaviour, i.e. RHP measures of preceding behaviours). Third, to quantify combined effects across sequential behaviours, we used composite levels of determination (*R*^*2*^_*C*_) and fractions of explained variance (FEV), based on the sums of the total variance exhibited by all measures (∑*TSS*) and the total variance explained by predictors across models (∑*ESS*). In order to test the effect of strategic decisions on agonistic motivation, we tested integrated effects on motivation from the same predictor categories, i.e. trait aggressiveness score, noise and nest, and RHP (weight and behaviour), sequentially (probe 1 and 2) and on average scores. Finally, we identified intuitive behavioural components using an agglomerative hierarchical clustering method - linking the closest variables and clusters at each step based on their absolute correlation (absolute value of Pearson’s coefficient, | *r* |) - and then quantified behavioural components from the correlation matrix (PCA: Bartlett’s, *P* < 0.001; *ρ* = 0.462 [34]) and tested whether normalised scores for each component were predicted by normalised scores of average motivation and its predicted fits and residuals from the model. This was in order to examine whether motivation influenced behavioural variation and if this was a mechanism for mediating strategic changes, i.e. whether factors influence behaviour by first influencing motivation.

## Supplementary Information

**Additional file 1: Table S1.** Statistical outputs for each model use to analyse behavioural measures and motivation probes.

**Additional file 2: Table S2.** Results of principal components analyses (PCA) of contest behaviour across the two mirror tests, day 4 and 7, used to quantify aggressiveness.

**Additional file 3:** Raw data and calculations.

## Data Availability

All data generated or analysed during this study are included in this published article and its supplementary information files.

## Abbreviations

RHP: Resource Holding Potential
PCA: Principal Components Analysis
FEV: Fraction of Explained Variance
