# Supplementary material for: Complex strategies: an integrative analysis of contests in Siamese fighting fish
Source: BMC Zool. 2022 Dec 9;7:59. doi: 10.1186/s40850-022-00156-3 (PMC10127297; doi:10.1186/s40850-022-00156-3)
Supplement: Supplementary file 1 — Additional file 1: Table S1. Statistical outputs for each model use to analyse behavioural measures and motivation probes. [file 40850_2022_156_MOESM1_ESM.docx]

| **Table S1.** Statistical outputs for each model use to analyse behavioural measures and motivation probes. | | | | | | | | | | |
| --- | --- | --- | --- | --- | --- | --- | --- | --- | --- | --- |
| **Behaviour** | | | | |  | **Motivation (Startle Recovery)** | | | | |
| Predictor | | *Χ^2^* | *P* | *β* |  |  | Predictor | *Χ^2^* | *P* | *β* |
| **Initiation: *R^2^* = 0.116** | | | | |  | **Probe 1: *R^2^* = 0.276** | | | | |
| Aggressiveness | | 191.42 | <0.001 | 0.390 |  | Trait | Aggressiveness | 1.91 | 0.167 | 0.061 |
| Resource  Value | Nest | 27.06 | <0.001 | - 0.336 |  | Resource | Nest | 14.37 | <0.001 | 0.440 |
|  | Noise | 98.72 | <0.001 | 0.594 |  |  | Noise | 10.88 | 0.001 | -0.343 |
|  | Interaction | 44.50 | <0.001 | -0.632 |  | Size | Focal | 17.09 | <0.001 | -1.547 |
| Size (g) | Focal | 1.68 | 0.195 | - 0.296 |  |  | Opponent | 34.14 | <0.001 | -2.735 |
|  | Opponent | 6.86 | 0.009 | - 0.739 |  |  | Interaction | 32.85 | <0.001 | -1.298 |
|  | Interaction | 4.05 | 0.044 | -0.272 |  | display | Focal | 3.02 | 0.082 | 0.124 |
| **Frontal Display: *R^2^* = 0.186** | | | | |  |  | Opponent | 0.82 | 0.364 | 0.097 |
| Aggressiveness | | 567.72 | <0.001 | 0.155 |  | Attack | Focal | 11.50 | 0.001 | 0.318 |
| Resource  Value | Nest | 424.86 | <0.001 | 0.317 |  |  | Opponent | 0.05 | 0.830 | 0.023 |
|  | Noise | 0.01 | 0.924 | -0.002 |  | Retreat | Focal | 0.83 | 0.362 | -0.080 |
|  | Interaction | 34.56 | <0.001 | -0.128 |  |  | Opponent | 0.19 | 0.659 | 0.052 |
| Size (g) | Focal | 0.68 | 0.409 | -0.042 |  |  | Interaction | 6.00 | 0.014 | 0.208 |
|  | Opponent | 27.63 | <0.001 | -0.336 |  |  | Air-breathing | 1.73 | 0.189 | 0.175 |
| Opponent Display | | 837.81 | <0.001 | 0.206 |  | **Probe 2: *R^2^* = 0.557** | | | | |
| **Lateral Display: *R^2^* = 0.353** | | | | |  | Trait | Aggressiveness | 17.11 | <0.001 | -0.102 |
| Aggressiveness | | 466.43 | <0.001 | -0.232 |  | Resource | Nest | 117.44 | <0.001 | 0.701 |
| Resource  Value | Nest | 7.66 | 0.006 | -0.068 |  |  | Noise | 16.53 | <0.001 | -0.227 |
|  | Noise | 55.85 | <0.001 | 0.181 |  | Size | Focal | 54.05 | <0.001 | -2.905 |
|  | Interaction | 113.85 | <0.001 | -0.367 |  |  | Opponent | 120.96 | <0.001 | -1.546 |
| Size (g) | Focal | 83.36 | <0.001 | 0.772 |  |  | Interaction | 107.86 | <0.001 | -1.332 |
|  | Opponent | 7.32 | 0.007 | 0.298 |  | display | Focal | 175.06 | <0.001 | 0.615 |
|  | Interaction | 30.43 | <0.001 | -0.283 |  |  | Opponent | 0.16 | 0.690 | 0.026 |
| Opponent Display | | 2194.04 | <0.001 | 0.705 |  | Attack | Focal | 84.85 | <0.001 | 0.491 |
| **Attack: *R^2^* = 0.116** | | | | |  |  | Opponent | 46.10 | <0.001 | -0.366 |
| Aggressiveness | | 2.94 | 0.086 | 0.525 |  | Retreat | Focal | 94.38 | <0.001 | -0.562 |
| Resource  Value | Nest | 6.67 | 0.010 | -1.227 |  |  | Opponent | 0.98 | 0.321 | -0.072 |
|  | Noise | 5.95 | 0.015 | -1.161 |  |  | Interaction | 4.03 | 0.045 | -0.1132 |
|  | Focal | 0.27 | 0.603 | 0.376 |  |  | Air-breathing | 4.89 | 0.027 | 0.179 |
|  | Opponent | 0.48 | 0.487 | 0.325 |  | **Mean: *R^2^* = 0.501** | | | | |
| Display  (Rate) | Opponent | 5.82 | 0.016 | -0.603 |  | Trait | Aggressiveness | 1.45 | 0.228 | 0.037 |
|  | Focal | 15.68 | <0.001 | 0.660 |  | Resource | Nest | 106.02 | <0.001 | 0.502 |
| Opponent Attack | | 4.67 | 0.031 | 1.301 |  |  | Noise | 28.75 | <0.001 | -0.258 |
| **Retreat: *R^2^* = 0.191** | | | | |  | Size | Focal | 30.56 | <0.001 | -1.451 |
| Aggressiveness | | 87.73 | <0.001 | -0.090 |  |  | Opponent | 70.99 | <0.001 | -2.758 |
| Resource  Value | Nest | 202.55 | <0.001 | -0.346 |  |  | Interaction | 64.87 | <0.001 | -1.285 |
|  | Noise | 81.91 | <0.001 | 0.215 |  | display | Focal | 163.02 | <0.001 | 0.435 |
|  | Interaction | 68.55 | <0.001 | 0.257 |  |  | Opponent | 3.13 | 0.077 | 0.080 |
| Size (g) | Focal | 2.32 | 0.128 | 0.113 |  | Attack | Focal | 22.03 | <0.001 | 0.308 |
|  | Opponent | 55.81 | <0.001 | 0.695 |  |  | Opponent | 1.79 | 0.181 | -0.091 |
|  | Interaction | 33.40 | <0.001 | -0.255 |  | Retreat | Focal | 73.39 | <0.001 | -0.403 |
| Display  (Rate | Opponent | 0.00 | 0.972 | -0.001 |  |  | Opponent | 1.61 | 0.204 | -0.074 |
|  | Focal | 562.53 | <0.001 | 0.271 |  |  | Air-breathing | 5.81 | 0.016 | 0.233 |
|  | Interaction | 333.20 | <0.001 | -0.102 |  |  |  |  |  |  |
| Attack  (Y/N) | Opponent | 13.40 | <0.001 | -0.104 |  |  |  |  |  |  |
|  | Focal | 13.70 | <0.001 | -0.080 |  |  |  |  |  |  |
|  | Interaction | 86.06 | <0.001 | 0.401 |  |  |  |  |  |  |
| Opponent Retreat | | 137.19 | <0.001 | 0.186 |  |  |  |  |  |  |
