# Supplementary material for: Complex strategies: an integrative analysis of contests in Siamese fighting fish
Source: BMC Zool. 2022 Dec 9;7:59. doi: 10.1186/s40850-022-00156-3 (PMC10127297; doi:10.1186/s40850-022-00156-3)
Supplement: Supplementary file 2 — Additional file 2: Table S2. Results of principal components analyses (PCA) of contest behaviour across the two mirror tests, day 4 and 7, used to quantify aggressiveness. [file 40850_2022_156_MOESM2_ESM.docx]

| **Table S2.** Results of principal components analyses (PCA) of contest behaviour across the two mirror tests, day 4 and 7, used to quantify aggressiveness. | | | | |
| --- | --- | --- | --- | --- |
|  |  |  |  |  |
|  | PCA Test 1 (day 4) | | PCA Test 2 (day 7) | |
|  | Loadings | Communality | Loadings | Communality |
|  |  |  |  |  |
| Initial latency (s) | -0.652 | 0.425 | -0.451 | 0.204 |
| Total display duration (s) | 0.494 | 0.244 | 0.900 | 0.810 |
| No. Attacks | 0.679 | 0.462 | 0.850 | 0.723 |
| *Eigenvalue* | *1.130*  *0.377* | | *1.736*  *0.579* | |
| *% variance* |  |  |  |  |
|  |  |  |  |  |
